# Flexible electronics research knowledge mapping based on VOSviewer and CiteSpace bibliometric analysis

**DOI:** 10.1186/s11671-026-04706-3

**Published:** 2026-06-09

**Authors:** Qing Li, Jingya Geng, Wen Zhou, Huamin Chen

**Affiliations:** 1 Minjiang University, Fuzhou, Fujian China; 2 Institute of Semiconductors, Chinese Academy of Sciences, Beijing, 100083 China; 3 Pingtan Yuxiang Times Technology Co., Ltd, Fuzhou, 350108 Pingtan County China

**Keywords:** Flexible electronics, Bibliometric analysis, VOSviewer, CiteSpace, Knowledge mapping, Web of science

## Abstract

Innovations in polymer chemistry and materials science have accelerated the development of high-performance flexible electronics for applications in various fields. While prior research has extensively reviewed progress within specialized subfields of flexible electronics, a systematic examination of the field's overall research landscape and its interconnections with adjacent knowledge domains remains absent. This review presents the first detailed bibliometric analysis of flexible electronics research trends, effectively balancing scholarly rigor with accessibility. Through VOSviewer and CiteSpace analysis of publications spanning 2006–2025, the publications have maintained steady growth with a markedly increased growth rate in recent years. Wei Huang was the most productive authors, while John A. Rogers showed the highest total link strength. Considering the productive and impactful institution, China and USA are unequivocally the preeminent forces in the field of flexible electronic. And South Korea and Japan remain significant forces that cannot be overlooked. Flexible electronic, performance, fabrication, films, graphene, transparent, and nanoparticles were among the high-frequency keywords. Four future development directions were recognized including material innovation, advanced manufacturing technology revolution, multimodal flexible integrated sensors, and machine learning-enhanced flexible sensors for health monitoring. This bibliometric investigation aims to provide foundational guidance and strategic direction for advancing flexible electronics research.

## Introduction

Flexible electronics refers to the large-scale integration of diverse material system and functional components onto flexible substrates, enabling the creation of stretchable/bendable flexible information devices and systems. It is characterized by its multi-disciplinary nature, integrating materials science, chemistry, mechanics, electronics, and computer science. Flexible electronics possess excellent characteristics such as light weight [1], adaptable form factors [2], and reconfigurable functionality [3], fundamentally altering the rigid physical form of traditional rigid electronics. Consequently, flexible electronics is considered to be a major development direction in chemistry and electronic field, which will greatly promote the development of health monitoring [4], human–machine interaction [5], metaverse [6] and the Internet of Things [7].

The concept of flexible electronics can be traced back to the research of organic electronics, which originated in the 1980s. The concept of stretchable inorganic-based flexible electronics was first proposed in 2006 by John A. Rogers at the University of Illinois and Huang Yonggang at Northwest University [8]. Functional materials commonly used in flexible electronic include flexible dielectric materials (such as Polydimethylsiloxane) [9, 10], flexible semiconductor materials (such as N-type hydrogel) [11, 12], flexible conductive materials (such as liquid metal) [13, 14], and flexible degradable materials (such as Polyvinyl Alcohol) [15, 16]. The progress in flexible electronics materials is fundamentally dependent on chemical contributions, as exemplified by the development of core materials (e.g., flexible substrates, organic semiconductors) and critical manufacturing processes (such as solution-based methods and interfacial chemistry). Unique structural designs including island-bridge structures [17, 18], wavy/serpentine structures [19], buckling-induced self-assembled structures [20], and strain-isolation structures [21], have enhanced the mechanical performance of flexible electronics. According to the functionality, the flexible electronics can be classified as flexible display devices [22, 23], flexible sensing devices [24, 25], flexible energy harvesting and storage devices [26, 27], and flexible circuits [28, 29].

In recent years, an increasing number of research groups have engaged in flexible electronics research especially in chemistry field, leading to significant advances across various aspects of the field. And numerous related review articles have published [30–33]. However, as flexible electronics is inherently interdisciplinary, involving electronic science and technology, materials science, mechanics, chemistry, and physics. A comprehensive consideration and analysis of its current research landscape and future development trajectories is urgently needed. However, to the best of our knowledge, there is almost no published research or review papers adopting bibliometric tools to quantitatively analyze this field. The application of bibliometric tools to quantitatively analyze flexible electronics provides a novel perspective that brings us new insights. Specifically, this perspective enables us to systematically and objectively depict the field’s overall research landscape and its interconnections with adjacent knowledge domains, which cannot be achieved by traditional qualitative reviews.

Bibliometric analysis serves as an effective tool for objectively examining the current state and developmental trajectory of scientific fields [34, 35]. This quantitative methodology leverages information extracted from published records, encompassing journals, publication dates, authors, institutions, affiliated countries, and keywords. However, manually extracting and systematically analyzing research trends becomes increasingly difficult as a field matures and its research output expands exponentially over years. Employing reliable analytical tools is therefore essential. Software platforms like VOSviewer and CiteSpace have gained widespread adoption across diverse academic disciplines, establishing bibliometric analysis as a prominent and frequently employed method for identifying developmental trends [36–39]. A key challenge, however, lies in navigating the explosive and complex growth of scientific and technological information: how to comprehensively and accurately synthesize this vast data while effectively extracting meaningful insights and balancing research depth with breadth.

In this work, a flexible electronics research knowledge mapping based on VOSviewer and CiteSpace bibliometric analysis was conducted to understand the state-of-the-art and emerging research directions. In Materials and Methods section, the definitive search protocol was established following successive query optimizations to delimit the research focus and exclude non-relevant records. The source data was collected from Web of Science (WOS) with 4244 articles. In the Results section, annual number of publications, distribution of journals, institutions, research countries/regions, and research authors were analyzed to identify the most productive and impactful items. Consequently, the co-occurrence of keywords and timeline visualization in flexible electronics were investigated to analyze the knowledge foundation, state-of-the-art and emerging research in flexible electronics, predicting the future development trend and possible challenges. Four primary future development vectors were recognized including material innovation, advanced manufacturing technology revolution, multimodal flexible integrated sensors, and machine learning-enhanced flexible sensors for health monitoring. In the Discussion section, we discussed the strength and limitations of the VOSviewer and CiteSpace bibliometric analysis-based review, aiming to provide useful insights for flexible electronics. In the Conclusion section, the main outcomes of the review were provided by clearly summarizing the research.

## Materials and methods

### Source database

**Fig. 1:**
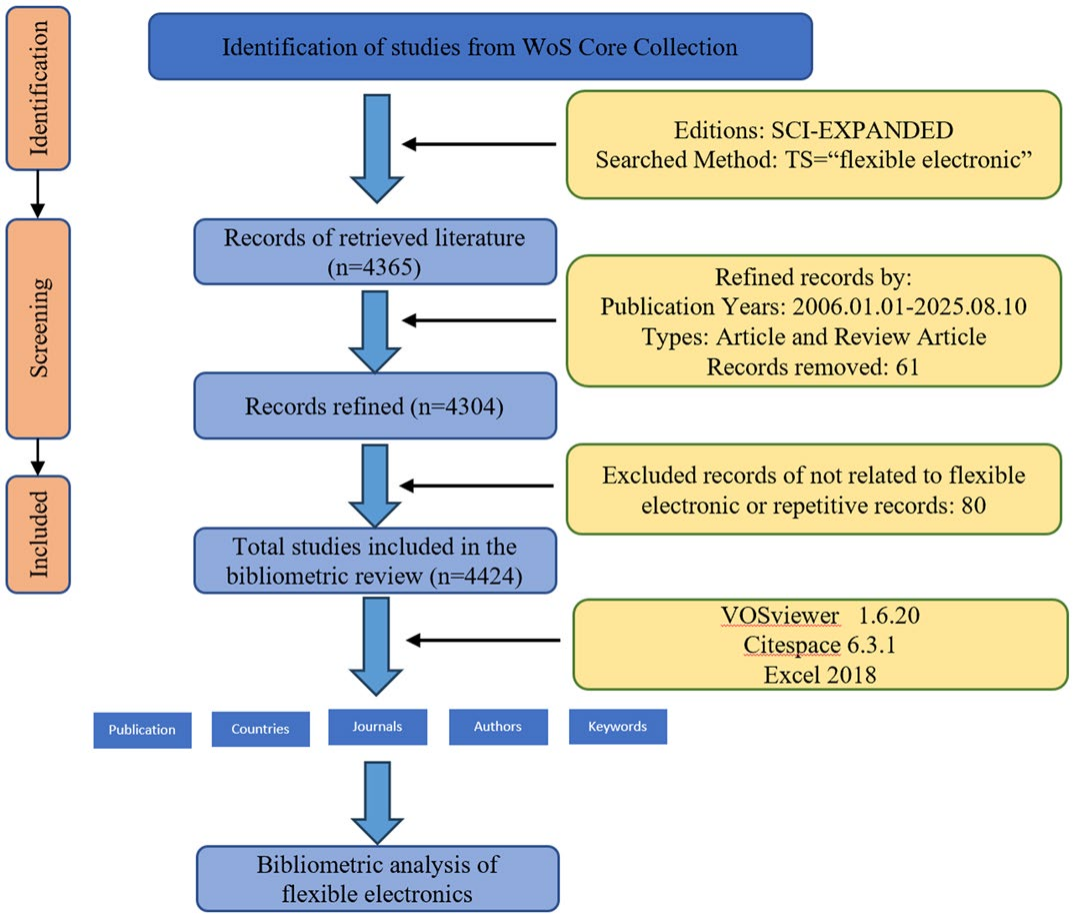
A flow chart of the data filtration process

The bibliometric approach has been employed to discuss statistical patterns in bibliography studies across various research fields over time. Data sources are fundamental, directly impacting the accuracy and reliability of the findings. WOS encompasses publications across various disciplines and is extensively utilized by researchers. Considering the research field and quality of publications, we collected publications from the WOS Science Citation Index Expanded (SCI-EXPANDED) in web of Science Core Collection for bibliometric analysis. Following successive query optimizations to delimit the research focus and exclude non-relevant records, the definitive search protocol was established. We conducted searches for the “flexible electronic” using double quotation marks in the topic field to enforce exact phrase matching, thereby eliminating lexical ambiguity and optimizing retrieval efficiency (4365 literatures). The study period spanned from 2006.01.01 to 2025.08.10 and the document type was restricted to “Article” and “Review Article”, as shown in Fig. 1. Subsequently, repetitive records or records of not related to flexible electronic were excluded. Finally, 4244 articles containing comprehensive information were analyzed to assess the state-of-the-art and emerging research directions in flexible electronic. The VOSviewer version is 1.6.20 and the Citespace version is 6.3.1.

### Bibliometric analysis

Bibliometric analysis is a research methodology that quantitatively analyzes literature information using mathematical and statistical techniques. Its purpose is to identify groups of researchers engaged in close collaboration or sharing common research interests. VOSviewer and CiteSpace softwares provide bibliometric analysis such as Co-authorship analysis and Co-occurrence analysis. Co-authorship is commonly used to analyze scholarly collaboration patterns. Its functions are to comprehend collaboration patterns/relationships, and to identify potential academic partners. Co-occurrence denotes the phenomenon where two or more projects appear simultaneously within the same dataset. It is frequently employed to analyze semantic relationships or dynamic network structures. Within the software settings, the size of the threshold influences the number of visualized nodes. A higher threshold results in fewer items meeting the filtering criteria, consequently reducing the number of visualized nodes. Node circles represent individual items. The size, color, and labeling of the circles reflect the weight or significance of the items. Lines connecting the nodes represent relationships between items. The thickness, color, and intensity of the lines depend on the strength of association between the connected items.

## Results

### Trend analysis of the number of publications

**Fig. 2:**
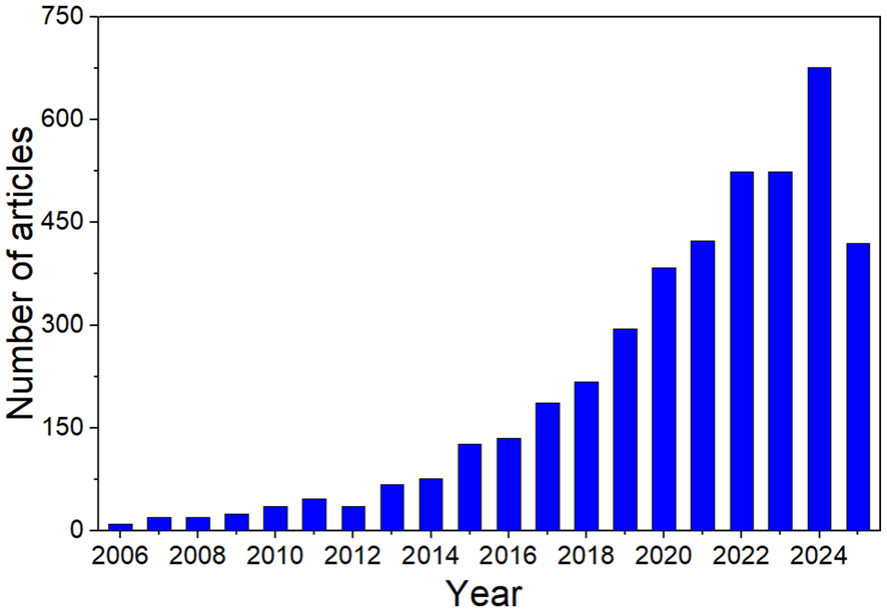
Trend of annual publications on flexible electronic from 2006 to 2025

This study identified 4244 publications on flexible electronic from the WOS database from 2006 to 2025. The chronological distribution of publications on flexible electronic is displayed in Fig. 2. Obviously, the publications show an exponentially growth trend with year and can be divided into three stages. In the first period (2006–2012), the annual number of publications is less than 50. In the second stage (2013–2021), the annual number of publications showed rapid growth due to the popularization of wearable electronics for personalized diagnosis and treatment. In this period, the flexible materials, manufacturing technologies and application exploration have made significant development. In the last stage (2022–2025), the annual publications surpassed 500, reaching 676 by 2024. The disruptive advancement of flexible electronic is principally driven by the accelerated integration of multidisciplinary knowledge domains and powerful market-driven imperatives.

### Journal distribution

The number of publications and the number of citations represent the impact of a focal journal. The “Bibliographic coupling” method was employed to analyze the “Sources” project, with the “Minimum number of documents of a source” set to 1, yielding results. The 4244 articles were published in 631 journals, with 54 journals each publishing more than 15 articles. As shown in Table 1, the top five journals were ACS Applied Materials & Interfaces (n = 240), Chemical Engineering Journal (n = 140), Advanced Functional Materials (n = 119), Journal of Materials Chemistry C (n = 80), and Journal of Materials Chemistry A (n = 73). These journals are predominantly dedicated to materials science and chemistry. From the aspect of citations, the top five highly cited journals were Advanced Materials (11,746 citations), Advanced Functional Materials (10,243 citations), ACS Applied Materials & Interfaces (9769 citations), ACS Nano (8662 citations), and Chemical Engineering Journal (5910 citations).

**Table 1:** The top 15 journals for publication in flexible electronic

Rank	Sources	Articles	Citations
1	ACS Applied Materials and Interfaces	240	9769
2	Chemical Engineering Journal	140	5910
3	Advanced Functional Materials	119	10,243
4	Journal of Materials Chemistry C	80	3041
5	Journal of Materials Chemistry A	73	3768
6	Advanced Materials	71	11,746
7	Nanoscale	65	4469
8	Applied Physics Letters	59	2739
9	ACS Nano	57	8662
10	Small	56	3496
11	Advanced Materials Technologies	52	1255
12	Journal of Materials Science	49	501
13	Scientific Reports	47	1515
14	Nanotechnology	46	1116
15	ACS Applied Polymer Materials	46	352

**Fig. 3:**
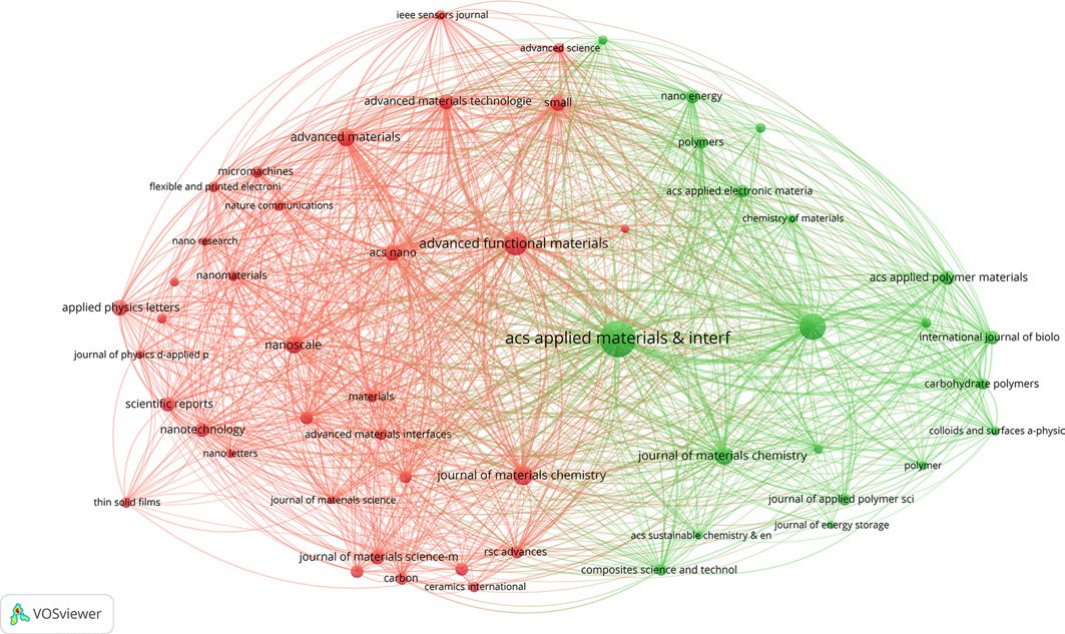
Bibliographic coupling map of the journal from 2006 to 2025

The distribution of journals can be categorized into two clusters, as shown in Fig. 3. Cluster 1 includes Advanced Materials, Advanced Functional Materials, Advanced Materials Technologies and ACS Nano (34 journals, depicted in red). Cluster 2 includes ACS Applied Materials & Interfaces, Chemical Engineering Journal, Journal of Materials Chemistry C and Journal of Materials Chemistry A (20 journals, depicted in green). Cluster 1 emphasizes materials and mechanism, and Cluster 2 focuses on chemistry and interfaces.

### Distribution of institutions

To identify leading research institutions in the field of flexible electronic, “co-authorship” method was used to analyze the “Organizations” project, with the “Minimum number of documents of an organization” set to 20, yielding 73 results. These institutions were used to construct a knowledge mapping network depicting cooperative relationships among research institutions in the field of flexible electronic. In this network, each circle represents a research institution, with the circle size indicating the number of documents produced by those institutions. The connection lines represent collaborative relationships with other institutions, with the line frequency corresponding to the degree of cooperation. Different colors mean distinct clusters of relationships.

A total of institutions was involved in flexible electronic research, with 30% of these institutions publishing only one article. The greatest number of published articles were from Chinese Academy of Sciences (312 publications). The top five research organizations are Chinese Academy of Sciences (n = 312), University of Chinese Academy of Sciences (n = 117), Tsinghua University (n = 111), South China University of Technology (n = 89), and Sichuan University (n = 83), as shown in Table 2. Furthermore, Average citations per publication were analyzed to assess the most infusive institutions. The top five institutions were The University of Tokyo (n = 215), Nanyang Technological University (n = 137), The Hong Kong Polytechnic University (n = 105), University of Illinois (n = 97) and Purdue University (n = 95). Combining the two indicators, we found that the institutes from China were more productive, while foreign institute were relatively impactful in the flexible electronic.

**Table 2:** The top 5 institutions in terms of number of publications in flexible electronic

Rank	Institution	Number of publications
1	Chinese Academy of Sciences	312
2	University of Chinese Academy of Sciences	117
3	Tsinghua University	111
4	South China University of Technology	89
5	Sichuan University	83

**Fig. 4:**
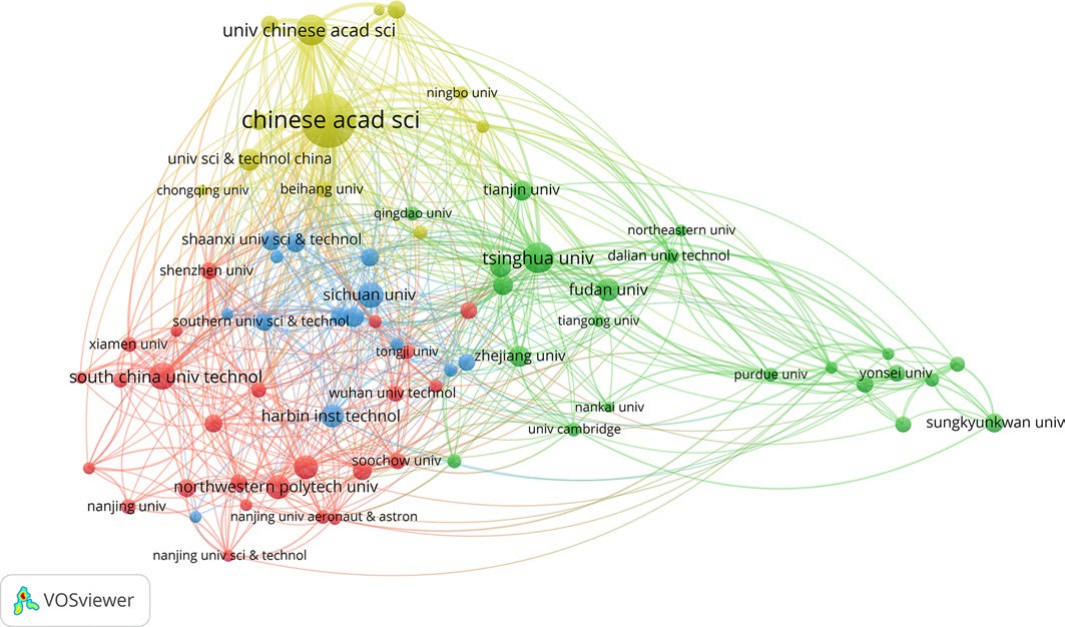
Cooperative knowledge map of the institutions from 2006 to 2025

There are four clusters displayed in Fig. 4. Cluster 1, 3 and 4 primarily consisted of Chinese institutions (depicted in red, yellow and blue). Cluster 2 included Korea University, Northwestern University, Purdue University and University of Illinois, predominantly representing American and Korea institutions (depicted in green). Interestingly, the clusters didn’t show geographical proximity, which reflected the interdisciplinary characteristics of flexible electronic.

### Distribution of research countries/regions

**Fig. 5:**
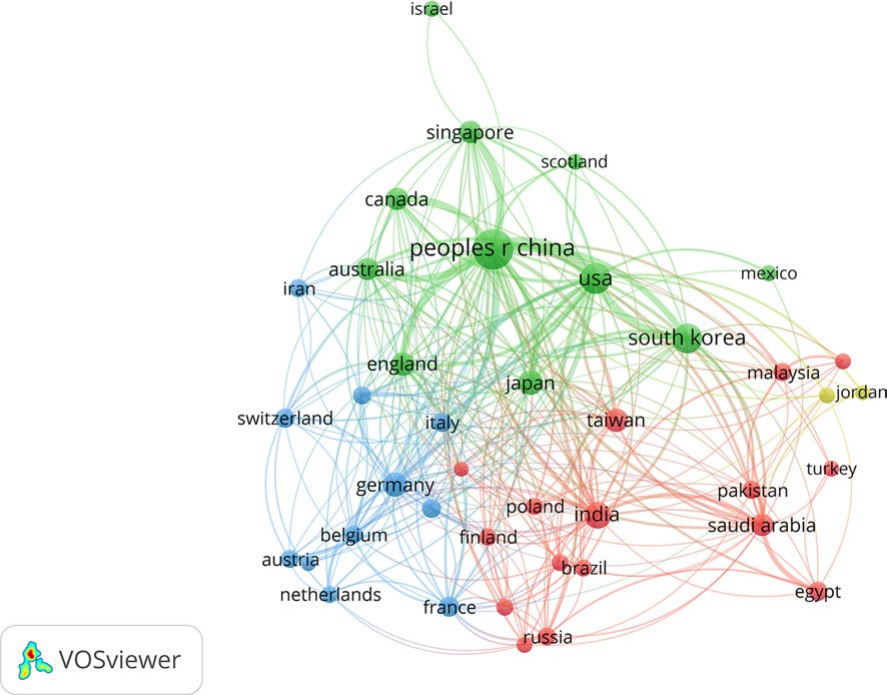
Cooperative knowledge map of the country/region from 2006 to 2025

“Co-authorship” method for the “Countries” project was adopted to analyze the leading position in the field of flexible electronic. “Minimum number of documents of a country” set to 1, yielding 86 results. The threshold was set to 10 to create the map, as shown in Fig. 5. The top five countries/regions were China (n = 2655), USA (n = 534), South Korea (n = 453), India (n = 230) and Japan (n = 142), as shown in Table 3. In addition to publications, the number of total citations of each country/region reflects the impact of a country/region in the field of flexible electronics. China, USA, South Korea, Germany and Japan were the most influential country/region. Considering the productive and impactful institution in Fig. 4, China and USA are unequivocally the preeminent forces in the field of flexible electronic. And South Korea and Japan remain significant forces that cannot be overlooked.

**Table 3:** The top 5 countries/regions in terms of number of publications in flexible electronic

Rank	Institution	Number of publications
1	China	2655
2	USA	534
3	South Korea	453
4	India	230
5	Japan	142

The frequency of collaboration between specific countries/regions can help us identify the cross-border collaboration. There are four clusters in the countries/regions co-authorship network. The cluster 1 includes India, Egypt and Saudi Arabia, which mainly contains Africa and western Asia. While cluster 2 contains the most impactful countries such as China, USA, South Korea and Japan. The third cluster “Europe” contains Germany, France, Italy and other European countries/regions. The last clusters only contain two countries of Jordan and United Arab Emirates. The four clusters all had cooperation with the other three clusters except themselves, demonstrating tightly interconnected global research networks in flexible electronic.

### Distribution of research authors

To identify the most impactful scholars in the flexible electronic, the “co-authorship” method for the “Authors” project was conducted. 44 authors have published more than 10 articles. The top 10 productive authors on flexible electronic were listed in Table 4. Huang Wei, hailed as the “Father of Flexible Electronics” in the industry, was the most productive authors. His research mainly focuses on organic electronics, plastic electronics, and flexible electronics disciplines. Considering from other parameters, Zhao Yue possessed the most citations of 1755 and John A. Rogers showed the highest total link strength, which also demonstrated their influence in flexible electronic.

**Table 4:** Top 10 authors in the field of flexible electronic

Rank	Sources	Articles	Citations
1	Huang Wei	22	1281
2	Wang Dong	20	421
3	Wang Wei	19	527
4	Li Yang	18	951
5	Zhang Wei	16	380
6	Liu Chuntai	15	910
7	Liu Jing	15	540
8	Chen Ying	14	795
9	Zhang Hao	14	439
10	Wang Cheng	14	623

**Fig. 6:**
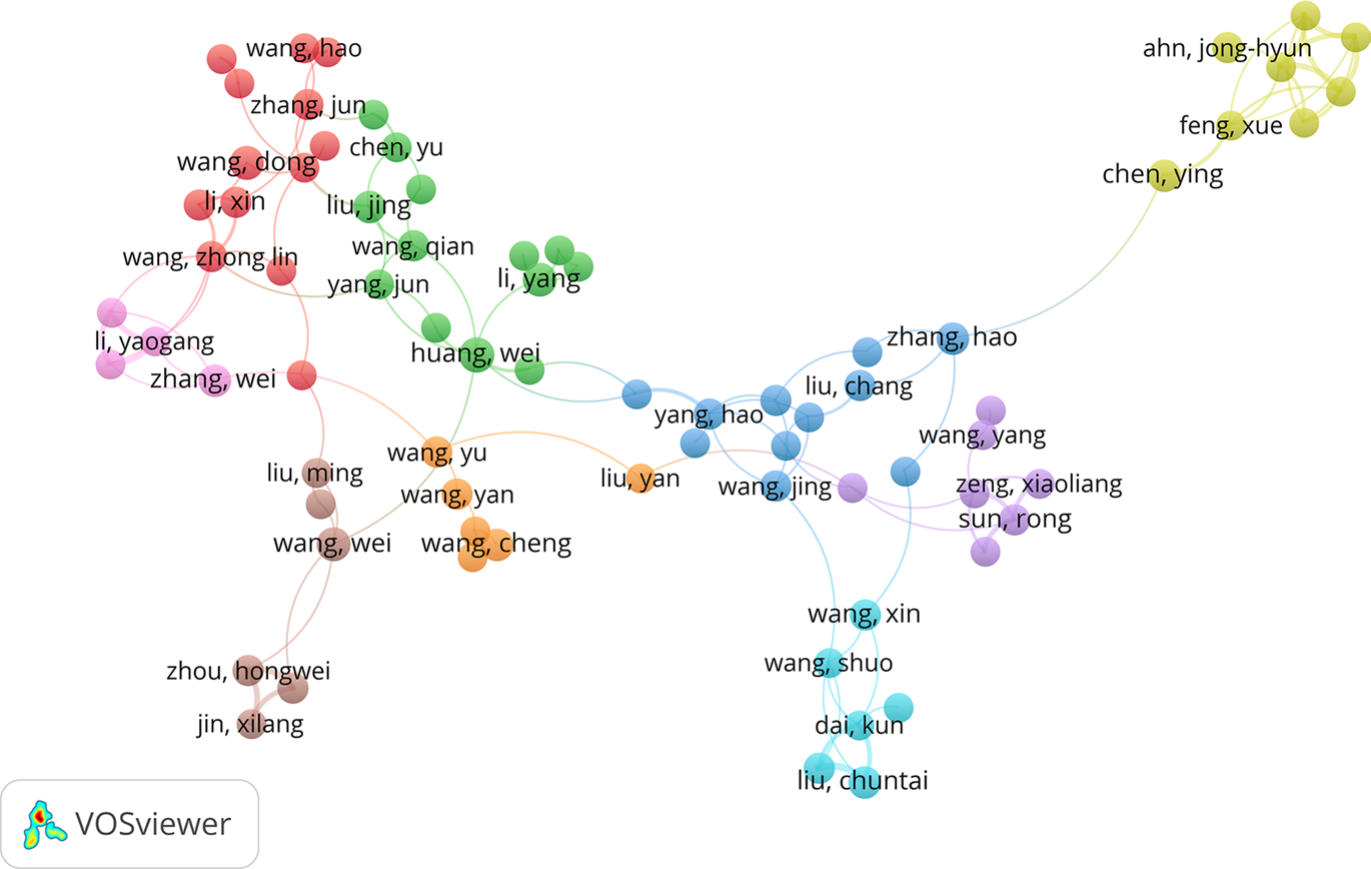
Co-authorship network map of authors in flexible electronic

There are 9 clusters exhibited in Fig. 6. The representative scholars included John A. Rogers, Huang Wei, and Wang Zhonglin. The 9 clusters have relatively clear boundaries between each cluster. This indicates that within the field of flexible electronic, there exists not only close collaboration but also intense competition. For example, the research teams of John A. Rogers and Huang Yonggang collaborate closely, with Huang focusing on mechanical simulations and Rogers on device fabrication. Except for their collaboration with the Feng Xue team at Tsinghua University, they do not have deep collaborations with other teams. This reflects distinct clusters (“circles”) and intense competition between them.

### Annual citation rate analysis

Comparisons of productivity and impact based on raw publication counts and total citations are biased by publication year, subfield differences, and document types. Therefore, annual average citation per paper was conducted, which effectively eliminates the deviation caused by publication year and subfield differences. The top ten annual citation rate were added in Table 5. Junwei Gu’ article demonstrated flexible (Fe_3_O_4_/PI)–Ti_3_C_2_Tx–(Fe_3_O_4_/PI) composite film with excellent EMI shielding performance, thermal conductivity, and mechanical properties, which has great potential for applications in EMI shielding protection for high-power, portable, and wearable flexible electronic devices, ranks first in terms of annual citation rate. Documents with high annual citation rates are widely recognized as landmark and classic studies within this domain.

**Table 5:** The top ten annual citation rate of flexible electronic

Rank	Title	Year	Average per year
1	Controlled Distributed Ti3C2Tix Hollow Microspheres on Thermally Conductive Polyimide Composite Films for Excellent Electromagnetic Interference Shielding	2023	248
2	Large-area ultrathin films of reduced graphene oxide as a transparent and flexible electronic material	2008	208.63
3	Stretching and Breaking of Ultrathin MoS2	2011	151.19
4	In-situ structure and catalytic mechanism of NiFe and CoFe layered double hydroxides during oxygen evolution	2020	143.86
5	Flexible Electronics toward Wearable Sensing	2019	133
6	Simple rules for the understanding of Heusler compounds	2011	129.63
7	Cellulose-Based Flexible Functional Materials for Emerging Intelligent Electronics	2021	115.71
8	Self-powered ultra-flexible electronics via nano-grating-patterned organic photovoltaics	2018	103.78
9	A Voltage-Boosting Strategy Enabling a Low-Frequency, Flexible Electromagnetic Wave Absorption Device	2018	102.11
10	Inkjet printing of single-crystal films	2011	99.88

### Co-occurrence network analysis

Keywords signify the article’s field and focus, directly mirroring its essential subject matter, thereby offering an indirect glimpse into the field's evolving trends. The high-frequency keywords and co-occurrence analysis are two fundamental bibliometric analysis.

**Fig. 7:**
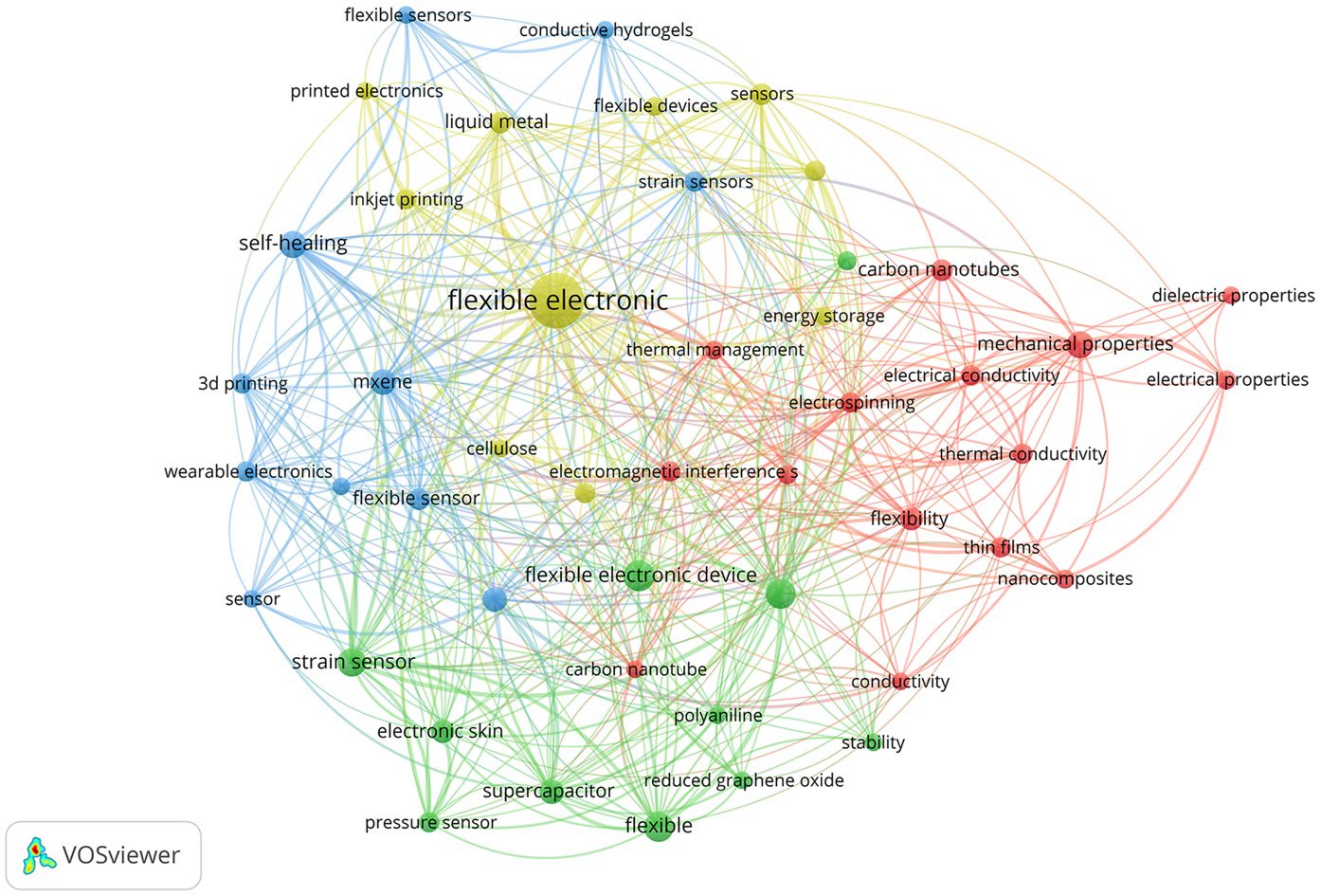
Co-occurrence network map of keywords in flexible electronic

“Co-occurrence” method was used to analyze the “Author keywords” project, with the “Minimum number of occurrences of a keyword” set to 60, yielding 77 results. There are 260 keywords with an occurrence frequency greater than 20, while 93 keywords greater than 50. The top 10 high-frequency keywords are flexible electronic, performance, fabrication, films, graphene, transparent, nanoparticles. The co-occurrence analysis of keywords in flexible electronic was displayed in Fig. 7. There are four clusters and each signified a research hotspot.

The red cluster mainly researched the properties of flexible materials including mechanical property, electrical property, thermal property and electromagnetic property. And the research object of these properties focused on carbon nanotubes. The green cluster mainly investigated the graphene-based flexible electronics such as electronic skin. Some flexible electronic devices such as strain sensor, pressure sensor, supercapacitor were included in these cluster. The third cluster explored emerging materials such as hydrogel and MXene-based flexible sensors. The hydrogel, especially conductive hydrogels and self-healing hydrogel, were promising flexible sensors. The last cluster includes flexible electronic, liquid metal, inkjet printing, printed electronics, silver nanowires, cellulose, sensor and energy storage. This cluster centered on printed flexible electronic by inkjet printing method.

**Fig. 8:**
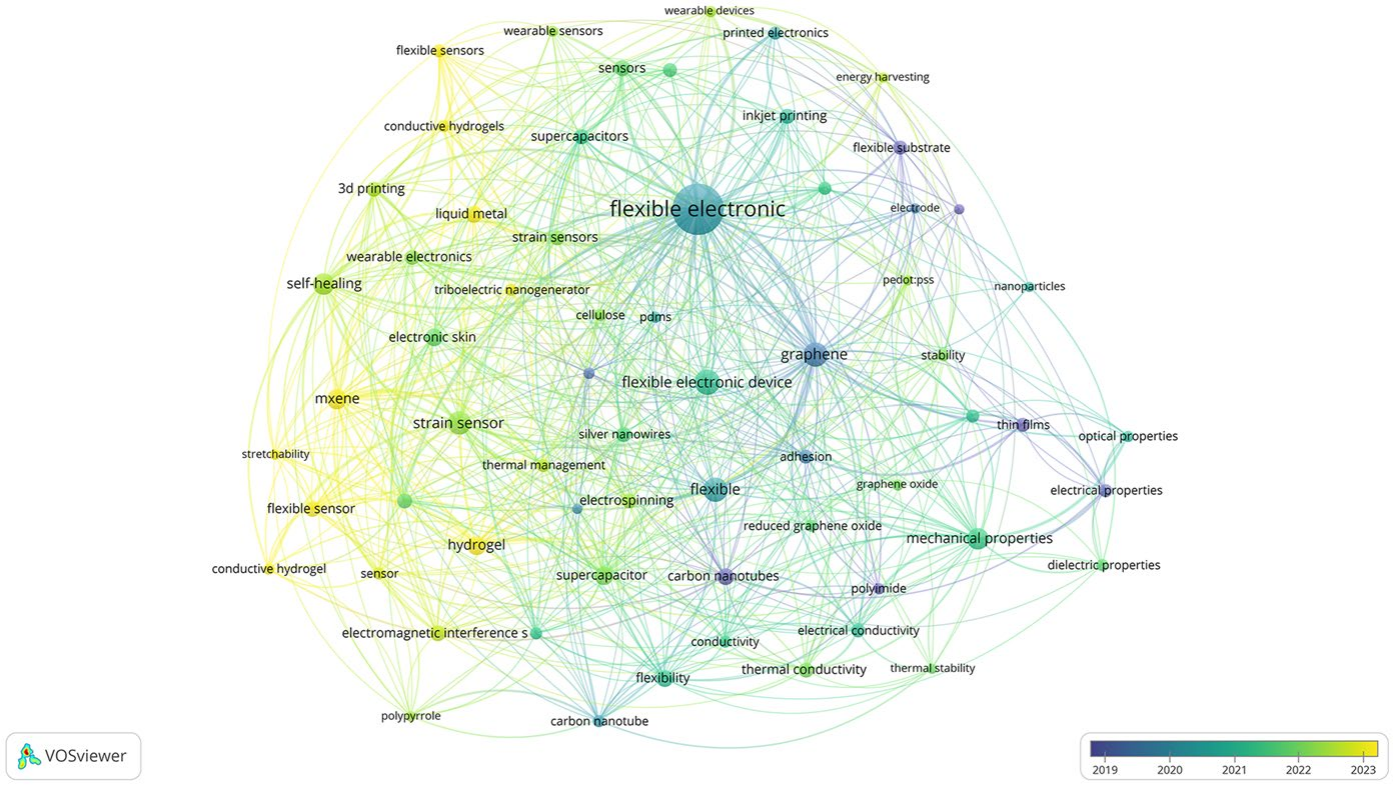
Timeline visualization of co-occurrence network map of keywords in flexible electronic

**Fig. 9:**
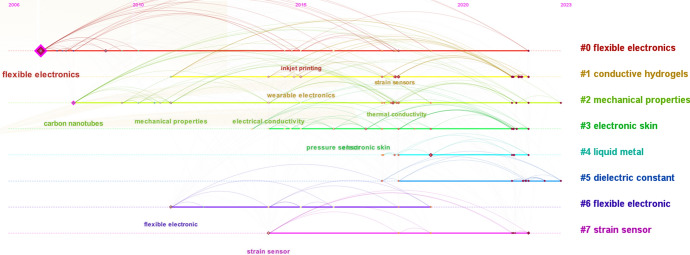
Evolution timeline diagram of keywords on flexible electronic

The timeline visualization of co-occurrence network map of keywords was conducted to analyze the development trend in flexible electronic. As shown in Fig. 8, the primary research focuses on the fabrication of flexible materials, including flexible substrate materials (PDMS and Polyimide) and flexible electrode materials (graphene, graphene oxide, PEDOT:PSS and silver nanowires). These studies explored various flexible electronics. In recent years, studies have shifted toward exploring the properties of carbon nanotube-based flexible electronics. The properties included electrical properties, mechanical properties, thermal properties, electromagnetic properties, and optical properties. Furthermore, the emerging materials such as conductive hydrogel, MXene and liquid metal attracted increasing attention due to its outstanding performance. 3d printing technology can also assist the fabrication of multimodal integrated devices. To further validate the development trend, the evolution timeline diagram was conducted by Citespace software. As shown in Fig. 9, there are 8 clusters including flexible electronics, conductive hydrogels, mechanical properties, electronic skin, liquid metal, dielectric constant, flexible electronic and strain sensor. The clusters in Fig. 9 are consistent with those in Fig. 7 such as flexible electronics, mechanical properties, conductive hydrogels and electronic skin. The research interest is now driven by the intrinsic properties of novel materials such as electrical properties and mechanical properties, enabling advances in multimodal sensor development.

## Keyword cluster analysis

**Fig. 10:**
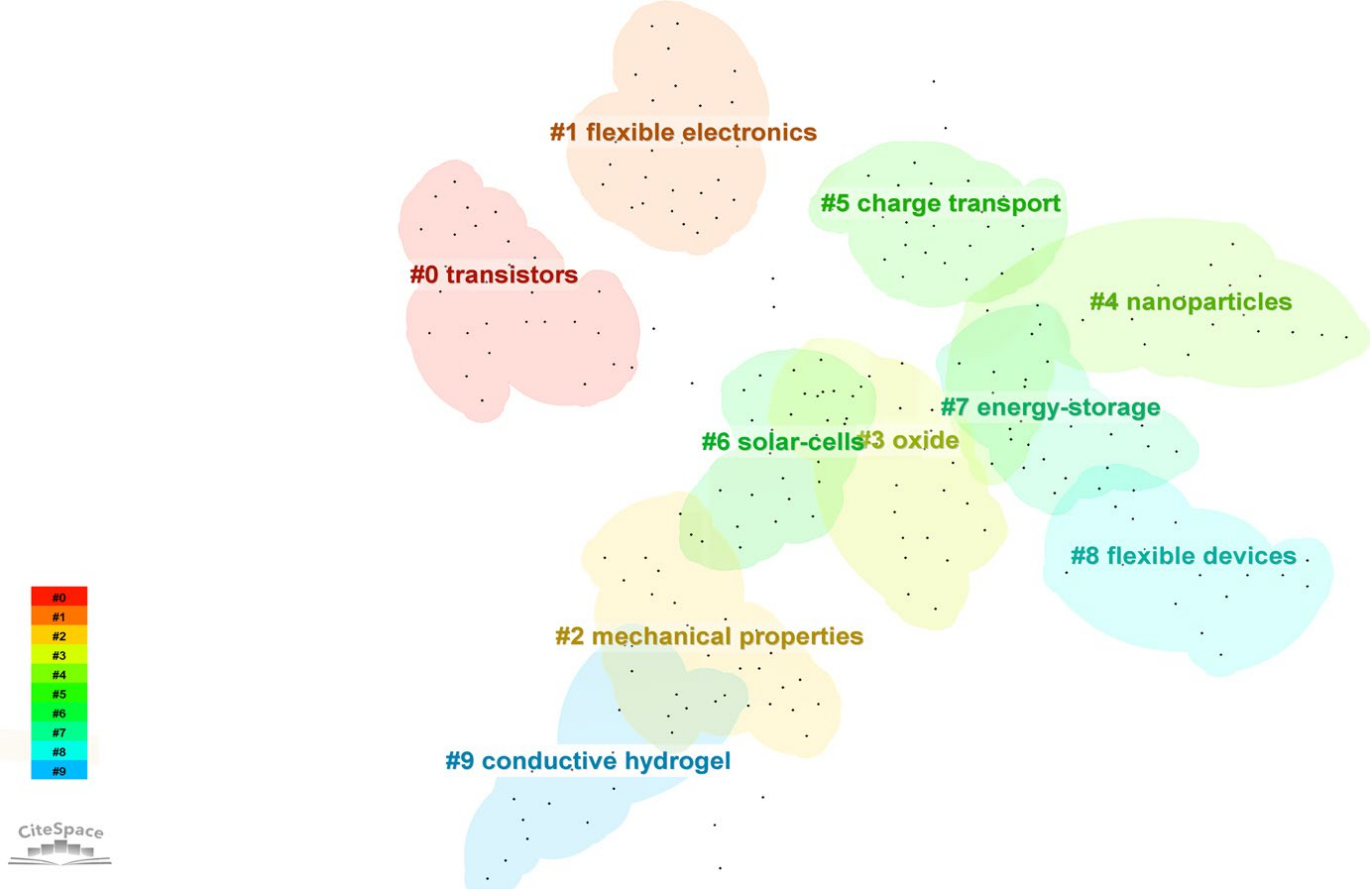
Keyword clustering analysis. Each distinct color represents a unique thematic cluster identified through the analysis

Citespace was used for keyword clustering analysis, yielding eight meaningful clusters (Fig. 10). The keyword clustering analysis of literatures in the field of flexible from 2006 to 2025 was conducted. The time slice was set to 3 years, the g-index with k = 10 was adopted for node screening, and the Pathfinder algorithm was used for network pruning. The results show that the modularity Q is 0.78 (> 0.3) and the silhouette score S is 0.91 (≥ 0.7), indicating that the clustering results are reliable. A total of 8 core clusters were formed: 0# transistors, 1# flexible electronics 2# mechanical properties, 3# oxide, 4# nanoparticles, 5# charge transport, 6# solar cells, 7# energy-storage, 8# flexible devices, 9# conductive hydrogel. These clusters collaboratively outline a comprehensive knowledge framework for flexible electronic, tracing a developmental trajectory from fundamental of mechanics, materials innovation, fabrication process to various flexible devices, demonstrating a clear translational pathway from basic research to commercialization that provides valuable guidance for future research directions.

## Reference burst detection

**Fig. 11:**
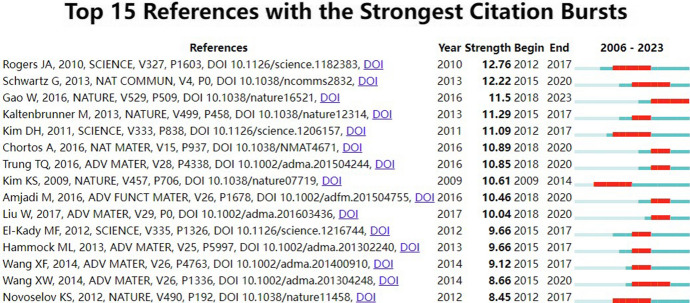
Top 15 references with the strongest citation burst in flexible electronic

Furthermore, we employed Citespace to perform a burst detection analysis on the reference list to identify highly cited literature experiencing surges in popularity. The top 15 references with the strongest citation burst in flexible electronic were listed in Fig. 11. A higher strength value means publications with greater influence, which represents its importance and critical role. Importantly, John A. Rogers, widely regarded as a founding father of flexible electronics, demonstrated the highest burst strength (strength = 12.76), with a citation burst period spanning from 2012 to 2017. This publication has become a methodological cornerstone in this field, which was frequently cited in publications. The study by Schwartz G. ranked second in burst strength (strength = 12.22). This study reported the fabrication of flexible pressure-sensitive organic thin film transistors with a maximum sensitivity of 8.4 kPa^−1^, a fast response time of < 10 ms, high stability over > 15,000 cycles and a low power consumption of < 1 mW. It demonstrated that the sensors can be used for non-invasive, high fidelity, continuous radial artery pulse wave monitoring, which may lead to the use of flexible pressure sensors in mobile health monitoring and remote diagnostics in cardiovascular medicine.

## Discussion

### Research growth

We conducted an extensive bibliometric analysis of research on flexible electronic from 2006 to 2025 using the WoS core collection database, focusing on key contributors, conceptual development, research hotspot and potential future research directions. The publications have maintained steady growth with a markedly increased growth rate in recent years (Fig. 2). This could be due to increased global attention, the gradual expansion of knowledge. As a highly interdisciplinary field, flexible electronics has entered a period of rapid development and presented a large-scale cooperation model, driven by the advancements in nanomaterials, artificial intelligence, micro-nano fabrication and other related fields, as well as the close collaboration between countries and institutions.

Comparisons of productivity and impact based on raw publication counts and total citations are biased by publication year, subfield differences, and document types. So top ten annual average citation per paper is analyzed in Table 5. This implies that publications in the early years introduced novel concepts and build a fundamental framework for flexible electronic. The later publications would have to cite these publications to increase its annual average citation per paper.

### Influential journals, institutions, countries/regions, and authors

From the aspect of citations, the top five highly cited journals were *Advanced Materials, Advanced Functional Materials, ACS Applied Materials & Interfaces, ACS Nano,* and *Chemical Engineering Journal*. The five journals are all among the most productive journals in the field of materials and chemistry. Although *ACS Applied Materials & Interfaces* and *Chemical Engineering Journal* had the highest number of publications (Table 1), the *Advanced Materials* and *Advanced Functional Materials* had the most citations. These journals have high impact factor, high H-index and high academic reputation. These results reveal that publications in journals with high impact factor are frequently cited.

In this study, the top five research institutions including Chinese Academy of Sciences, University of Chinese Academy of Sciences, Tsinghua University, South China University of Technology, and Sichuan University are based in China, indicating China’s institutional dominance. However, no Chinese institutions are included among the most influential institutions. It reveals that the institutes from China were more productive, while foreign institute were relatively impactful in the flexible electronic.

China, USA, South Korea, India and Japan are globally ranked as the top 5 countries leading in flexible electronic research. This is also consistent with the developmental history of this field. The United States, Japan and South Korea pioneered its early development, after which China and India have achieved rapid catch-up. As shown in Fig. 5, the four clusters all had cooperation with the other three clusters except themselves, demonstrating tightly interconnected global research networks in flexible electronic.

Huang Wei, hailed as the “Father of Flexible Electronics” in the industry, was the most productive authors, while John A. Rogers showed the highest total link strength, which also demonstrated their influence in flexible electronic. As shown in Fig. 6 and Table 4, the top productive authors in this field were all Chinese. The 9 clusters have relatively clear boundaries between each cluster. This indicates that within the field of flexible electronic, there exists not only close collaboration but also intense competition.

### Hotspot analysis

The top high-frequency keywords are flexible electronic, performance, fabrication, films, graphene, transparent, and nanoparticles. The co-occurrence analysis of keywords in flexible electronic was displayed in Fig. 7. According to the keyword co-occurrence cluster mapping and density visualization, there are four clusters and each signified a research hotspot. The main research topics in flexible electronic can be summarized into three aspects.

1. As the red and green cluster exhibited in Fig. 7, 2D materials and stretchable materials such as graphene, MXene and hydrogel has become the research hotspot of flexible electronics. These materials can be used to fabricate flexible displays, flexible sensors and flexible integrated circuits.
2. Mechanical properties are, to some extent, the most fundamental and critical performance metrics, as they provide essential guidance for the development of stretchable electronics and failure analysis under strain.
3. Flexible sensors, especially strain sensor, have been widely used in flexible electronic field for its simple structure, low cost, high stability and extensive utility. For example, numerous startups focused on flexible sensor technologies have emerged, such as the company founded by Professor Guo Chuanfei from Southern University of Science and Technology. These efforts are steadily advancing toward industrialization.

### Future research directions

According to the keywords co-citation networks and reference co-citation networks in Figs. 8, 9, 10 and 11, the development trend in flexible electronic could be predicted.

#### Material innovation

As the #1 conductive hydrogels and #4 liquid metal showed in the evolution timeline diagram of keywords, materials is the foundation of flexible electronics. Development of novel flexible/stretchable materials such as liquid metals, hydrogels, and conductive polymers can significantly enhance the performance of flexible electronics. Key breakthroughs include self-healing materials (restoring conductivity after damage), biodegradable electronics (eco-friendly solutions for implantable devices), and ultra-stretchable material (e.g., liquid metals and hydrogels) rely on polymer Chemistry and materials science. For example, Deng Tao from Shanghai Jiaotong University demonstrated the use of liquid metals with the integration of spacers, which show both metallic and fluidic properties, as stretchable hermetic seals [40]. Bao Zhenan from Stanford University reported a skin-interfacing hydrogel electrodes capable of on-demand adhesion and detachment [41].

#### Advanced manufacturing technology revolution

Overcoming limitations of traditional photolithography processes is important for fabricating flexible electronics with low price, large scale, high resolution and outstanding integration. Critical technologies encompass large-area roll-to-roll printing, multi-material 3D printing (such as the photocurable multi-material printing technology), laser-induced graphene, and inkjet printing technology. For example, Gao Wei reported a 3D-printed epifluicid electronic skin for machine learning-powered multimodal health surveillance [42]. Lin Yuan from University of Electronics Science and Technology of China introduced a skin-attachable, reprogrammable, multifunctional, adhesive device patch fabricated by simple and low-cost laser scribing of an adhesive composite with polyimide powders and amine-based ethoxylated polyethylenimine dispersed in the silicone elastomer [43].

#### Multimodal flexible integrated sensors

Although significant breakthroughs have been made in the research of single-mode and dual-mode flexible sensors, their application scope is still limited by their inability to meet the strict requirements of precise disease diagnosis in the medical and health field, as well as the practical needs of recognizing complex and variable stimuli in human motion. In view of this, researchers have begun to focus on the development of multimodal flexible sensors [44, 45] (pressure, strain, temperature, humidity, biochemical signals) in order to further break through technological bottlenecks. Multimodal sensors not only inherit the advantages of single and dual mode sensors, but also innovatively introduce a third or more stimulus response functions on this basis, greatly enriching the detection dimensions of sensors and improving their application flexibility and accuracy in multiple scenarios. This innovation not only broadens the application areas of sensors, but also brings unprecedented opportunities and challenges to fields such as medical health and sports monitoring.

#### Machine learning-enhanced flexible sensors for health monitoring

The flexible sensors enable long-term real-time monitoring. The processing of large amounts of data imposes a computing power barrier for practical application. Fortunately, the emerging of artificial intelligence (AI) seems to provide a solution, on account of its powerful signal processing and data analysis capabilities. AI is an important branch of computer science, whose core is to enable computers to complete complex work tasks like humans. Typically, machine learning (ML) is widely adopted to process massive regular data [46, 47]. ML method can learn rules and automatically build optimized models to solve problems after repeated training. Its prominent advantage is that it does not require the user to understand the inherent logic between features and results.

### Strengths and limitations

This study is the first to analyze research trends in flexible electronics in detail through a bibliometric analysis, which balances professionalism and readability. However, it has the following limitations. First, our literature search was strictly limited to specific keywords and the SCIE database. While this ensured the relevance and authority of the included publications, it may have excluded pertinent literature that was not highly relevant or indexed outside the SCIE database. Second, VOSviewer-based bibliometric analysis and visualization are subject to researcher bias via parameter configuration, potentially introducing subjectivity into the findings. Finally, we note that flexible electronics is an interdisciplinary field. Consequently, authors, particularly those in biology-related disciplines, often employ more specific keywords in their publications and may not explicitly mention “flexible electronics.” Notable examples include Professor Zhenan Bao (Stanford University) and Professor Wei Gao (California Institute of Technology).

## Conclusions

This study used bibliometrics to analyze literatures published from 2006 to 2025 on the flexible electronic. It focused on publication trends, journal distribution, institution distribution, research countries/regions distribution, relevant authors and co-occurrence network analysis, offering insights into the evolution of research hotspot and future research direction of flexible electronic. The publications have maintained steady growth with a markedly increased growth rate in recent years. Wei Huang was the most productive authors, while John A. Rogers showed the highest total link strength. Considering the productive and impactful institution, China and USA are unequivocally the preeminent forces in the field of flexible electronic. And South Korea and Japan remain significant forces that cannot be overlooked. Flexible electronic, performance, fabrication, films, graphene, transparent, and nanoparticles were among the high-frequency keywords. Four future development directions were recognized including material innovation, advanced manufacturing technology revolution, multimodal flexible integrated sensors, and machine learning-enhanced flexible sensors for health monitoring. As a highly interdisciplinary field, it is recommended that more collaboration among countries/regions and institutions should be encouraged.

## Data Availability

Data sharing is not applicable to this article, as no datasets were generated or analyzed in the current study.
